# Two Drinking Water Outbreaks Caused by Wastewater Intrusion Including Sapovirus in Finland

**DOI:** 10.3390/ijerph16224376

**Published:** 2019-11-09

**Authors:** Ari Kauppinen, Tarja Pitkänen, Haider Al-Hello, Leena Maunula, Anna-Maria Hokajärvi, Ruska Rimhanen-Finne, Ilkka T. Miettinen

**Affiliations:** 1 Department of Health Security, Expert Microbiology Unit, Finnish Institute for Health and Welfare, 70701 Kuopio, Finland; tarja.pitkanen@thl.fi (T.P.); anna-maria.hokajarvi@thl.fi (A.-M.H.); ilkka.miettinen@thl.fi (I.T.M.); 2 Department of Health Security, Expert Microbiology Unit, Finnish Institute for Health and Welfare, 00271 Helsinki, Finland; haider.al-hello@thl.fi; 3 Department of Food Hygiene and Environmental Health, Faculty of Veterinary Medicine, University of Helsinki, 00014 Helsinki, Finland; leena.maunula@helsinki.fi; 4 Department of Health Security, Infectious Diseases Control and Vaccinations Unit, Finnish Institute for Health and Welfare, 00271 Helsinki, Finland; ruska.rimhanen-finne@thl.fi

**Keywords:** waterborne outbreak, enteric viruses, contamination, drinking water, wastewater, sapovirus, microbial source tracking, fecal indicators, *Dientamoeba fragilis*

## Abstract

Drinking water outbreaks occur worldwide and may be caused by several factors, including raw water contamination, treatment deficiencies, and distribution network failure. This study describes two drinking water outbreaks in Finland in 2016 (outbreak I) and 2018 (outbreak II). Both outbreaks caused approximately 450 illness cases and were due to drinking water pipe breakage and subsequent wastewater intrusion into the distribution system. In both outbreaks, the sapovirus was found in patient samples as the main causative agent. In addition, adenoviruses and *Dientamoeba fragilis* (outbreak I), and noroviruses, astroviruses, enterotoxigenic and enterohemorragic *Escherichia coli* (ETEC and EHEC, respectively) and *Plesiomonas shigelloides* (outbreak II) were detected in patient samples. Water samples were analyzed for the selected pathogens largely based on the results of patient samples. In addition, traditional fecal indicator bacteria and host-specific microbial source tracking (MST) markers (GenBac3 and HF183) were analyzed from water. In drinking water, sapovirus and enteropathogenic *E. coli* (EPEC) were found in outbreak II. The MST markers proved useful in the detection of contamination and to ensure the success of contaminant removal from the water distribution system. As mitigation actions, boil water advisory, alternative drinking water sources and chlorination were organized to restrict the outbreaks and to clean the contaminated distribution network. This study highlights the emerging role of sapoviruses as a waterborne pathogen and warrants the need for testing of multiple viruses during outbreak investigation.

## 1. Introduction

The drinking water contaminated with pathogenic microbes may cause large community outbreaks with up to thousands of illness cases in both developing and developed countries. Several factors may cause a drinking water outbreak. Raw water contamination, treatment deficiencies, and distribution network failure are among the most common causes [1]. In addition, waterborne outbreaks have been associated with climatic conditions, especially with increased precipitation and heavy rainfall events [1,2,3,4]. The source of the contamination is most commonly wastewater which may harbor a large number of diverse pathogenic microbes.

In Finland, a food and waterborne outbreak surveillance system has revealed several waterborne outbreaks every year since 1997. In these outbreaks, norovirus has been the most common causative agent followed by *Campylobacter* [5,6]. In addition to noroviruses, the potential waterborne spread of other enteric viruses, such as adenoviruses [7,8], sapoviruses [9,10], enteroviruses [8], astroviruses [11] and rotaviruses [8] have been reported in Finland.

Sapoviruses are close relatives to noroviruses and the clinical symptoms of sapovirus gastroenteritis are indistinguishable from those caused by noroviruses. Though, in general, the clinical severity of sapovirus-associated disease is milder than that for norovirus and rotavirus [12]. Sapoviruses are common in wastewater [13,14], and due to the availability of improved methodologies, these viruses are also now being analyzed and detected more often. An increasing number of reports related to outbreaks and sporadic cases caused by sapovirus have been described, highlighting the emerging role of sapoviruses as a public health concern [15,16,17,18,19,20,21].

Traditionally, the microbiological quality of drinking water has been estimated by using fecal indicator bacteria (FIB), such as *Escherichia coli*, intestinal enterococci and *Clostridium perfringens*. These FIB are part of the normal flora in the intestinal tract of humans and other warm-blooded animals, and thus they are consistently present in wastewater. However, the capability of these indicators to measure water quality and predict waterborne outbreaks has been questioned [22,23,24]. Therefore, more specific and sensitive fecal indicators of water quality have been explored. Potential candidates are the genetic markers from the group of *Bacteroidales*, such as general *Bacteroidales* genetic marker (GenBac3) [25] and the host-specific HF183 marker [26], used as targets in quantitative PCR (qPCR) assays for the detection of fecal contamination and human wastewater pollution, respectively. Although the qPCR assays are often designed to target the ribosomal RNA gene (rDNA), it has been proven that the detection frequency of fecal bacteria in water can be enhanced by targeting the assays to rRNA transcripts instead of rDNA [27,28]. While *Bacteroidales* assays are widely applied in studies of microbial source tracking (MST) in surface waters [29], their use as part of community-wide waterborne outbreak investigations is rare [10]. Thus, more data to assess the suitability of these new indicators as a tool to describe drinking water contamination episodes, to detect drinking water quality deficiencies and their application in processes securing good drinking water quality, is needed.

This study describes two waterborne outbreaks both caused by the intrusion of wastewater into a drinking water distribution system due to pipe breakage. Causative agents of outbreaks were determined through investigations of patient and water samples and the suitability of both traditional FIB and new candidates (GenBac3 and HF183) to provide water quality information was evaluated.

## 2. Materials and Methods 

### 2.1. Outbreak Descriptions and Samples

This study describes two drinking water outbreaks in Finland in October 2016 (outbreak I) and January 2018 (outbreak II). Both outbreaks were initially caused by the drinking water pipe breakage and subsequent wastewater intrusion into the distribution system. Information regarding the outbreaks was collected from the local investigation reports, including retrospective questionnaires, and personal communications. The outbreaks were defined as waterborne outbreaks with a strong strength of association based on classification criteria presented previously [30,31].

#### 2.1.1. Outbreak I

**Figure 1:**
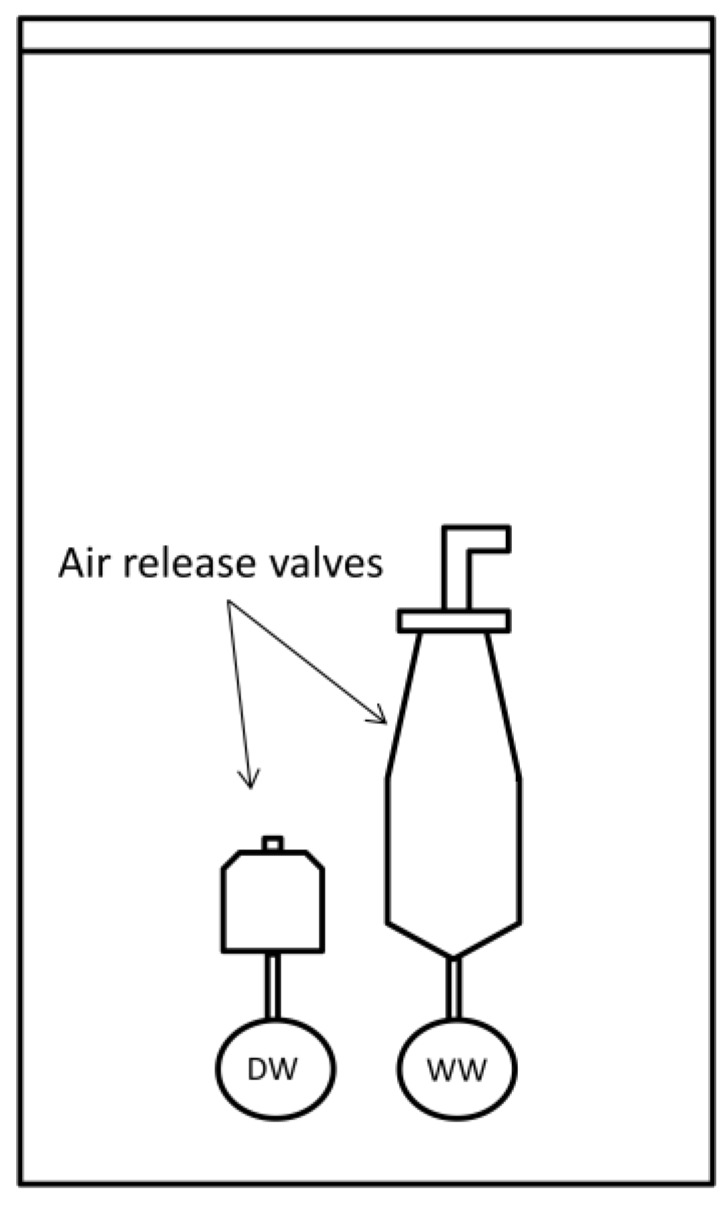
Schematic presentation of the maintenance well containing air release valves of drinking water (DW) pipe and wastewater (WW) pipe.

In outbreak I, the cause of the contamination was a maintenance well containing the air release valves of both drinking water and wastewater pipes (Figure 1). The air release valve of the wastewater pipe allowed wastewater to leak and accumulate into the maintenance well. Due to pipe breakage on the road construction site on 12th October 2016, the under pressure in the drinking water network caused the wastewater inflow from the maintenance well through the air release valve into the drinking water distribution system. The pipe breakage was detected and repaired immediately but the cross-contamination in maintenance well was detected only after six days on 18th October 2016.

Drinking water originating from the groundwater source was flocculated with KMnO_4_, pH was adjusted with NaOH followed by clarification and sand filtration through three sand basins and finally UV-treated prior to distribution. Drinking water was not chlorinated. Mitigation actions during the outbreak included the boil water advisory for two months (from 16th October to 16th December 2016) and chlorination for 50 days (from 16th October to 5th December 2016). The target chlorine levels were as follows: first 2 mg/L for 3 days, then 4.5 mg/L for 3 days and finally 1 mg/L for 44 days. In addition, alternative water sources were arranged for the water users during the outbreak. 

**Figure 2:**
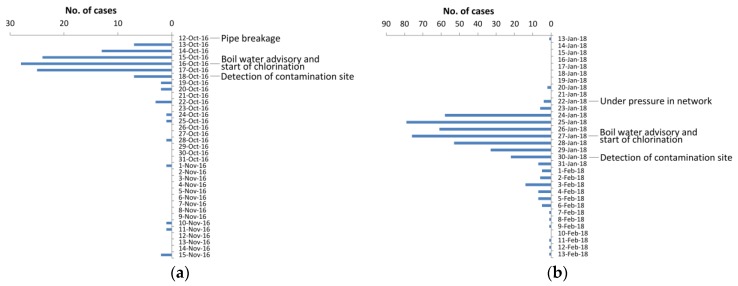
Epidemic curves in (**a**) outbreak I and (**b**) outbreak II, including key actions.

The drinking water contamination affected approximately 790 people. In order to estimate the magnitude of illness, questionnaires were sent to the households of the contaminated area. The response rate was 62% (294/471 households). In the analysis, only one response per household was included. Thus, in total, 115 symptomatic cases of 283 respondents were observed (Figure 2a). When respondents’ family members with gastrointestinal illness were taken into account, the estimated number of patients was 458. According to a questionnaire study, the first patients appeared one day after the pipe breakage. The median duration of the symptoms was one to two days and the most frequently reported symptoms included abdominal pain (94%, 101/107), nausea (91%, 100/110), diarrhea (89%, 100/112), abdominal swelling (83%, 86/104), muscular pain (66%, 64/97), vomiting (53%, 52/98) and fever (46%, 42/91). The symptoms suggested a viral point source outbreak with a rapid increase of cases followed by a fast decrease after the mitigation actions (Figure 2a). In the acute phase of the outbreak, stool samples were collected from patients between 19th October and 3rd November 2016, and were analyzed in clinical laboratories with routine tests for enteric viruses, pathogenic bacteria, and protozoans (Table 1).

FIB (*E. coli*, coliform bacteria, intestinal enterococci, and *C. perfringens*) were analyzed in local environmental laboratories from the water samples collected between 15th October 2016 and 27th January 2017. Drinking water samples were taken for pathogen analyses only after start of the chlorination on 24th October 2016 (n = 1) and 26th October 2016 (n = 3). Pathogen analyses for the water samples were selected and prioritized based on results from patients taking into account the available water volume. The early water samples were analyzed only for sapoviruses and protozoans (*Cryptosporidium* spp., *Giardia lamblia, Entamoeba histolytica,* and *Dientamoeba fragilis)*. On 28th November 2016, a raw water sample (dead-end ultrafiltration, DEUF), drinking water samples (n = 3, DEUF), biofilm samples from water meters (n = 9) and a sample from the contamination site (maintenance well) were collected and analyzed for sapo- and adenoviruses, MST markers (GenBac3 and HF183), *E. coli*, coliform bacteria, *C. perfringens* and protozoans. Moreover, a sewage sample from the municipal wastewater treatment plant was collected on 26th October 2016 and analyzed for sapoviruses and protozoans.

#### 2.1.2. Outbreak II

In outbreak II, both a drinking water pipe and a wastewater pipe were broken at the same site. It was suggested that the drinking water pipe had leaked for several months near the wastewater pipe, and eventually, this caused a collapse of the waterlogged soil and the breakage of the sewer. The under pressure event in the drinking water distribution system during the search of the leakage on 22nd January 2018 most probably caused the inflow of wastewater from the contamination site into the drinking water network. The contamination site with broken pipes was detected eight days after the assumed contamination event on 30th January 2018.

Drinking water originating from the groundwater source was alkalized and UV-treated prior to distribution. Drinking water was not chlorinated. Mitigation actions during the outbreak included the boil water advisory for four weeks (from 27th January to 23rd February 2018) and chlorination for six weeks (from 27th January to 10th March 2018) with chlorine levels ranging from <1 mg/L–2 mg/L and including 3–5 days intensive chlorination with chlorine levels 5 mg/L–10 mg/L (started on 6th February 2018). In addition, alternative water sources were arranged for six weeks (from 29th January to 11th March 2018). 

The drinking water contamination affected approximately 4000 people. During January–February, 463 persons with gastrointestinal illness contacted local primary health care. Some cases occurred already before the assumed under pressure event, but most of the patient cases appeared from 24th January–30th January 2018 (Figure 2b). Symptoms lasted on average for two days and included diarrhea (76%, 352/463), vomiting (65%, 299/463) and fever (32%, 150/463). Stool samples were collected during the acute phase of the outbreak and were analyzed in clinical laboratories with routine tests for enteric viruses, pathogenic bacteria, and protozoans (Table 1). Since the clinical laboratory method did not distinguish between norovirus genogroups, seven samples were further analyzed by the genogroup-specific real-time RT-PCR [32].

FIB (*E. coli*, coliform bacteria, intestinal enterococci, and *C. perfringens*) were analyzed from drinking water samples collected between 27th January 2018 and 5th March 2018 in a local environmental laboratory. Drinking water samples for pathogen (sapo-, noro- and adenovirus, pathogenic *E. coli* strains, *Campylobacter* spp., *Giardia* spp. and *Cryptosporidium* spp.) and MST marker (GenBac3 and HF183) analyses were taken before chlorination on 27th January 2018 (n = 1), after the initial low level chlorination (<1 mg/L) on 29th January 2018 and 6th February 2018 (n = 2) and after the intensive chlorination on 14th February 2018 (n = 3, DEUF). A surface water sample from the contamination site and a biofilm sample from water meter were collected on 31st January and were analyzed for the selected microbes (Table 2).

### 2.2. Environmental Investigation

#### 2.2.1. Sample Collection and Concentration

Water was collected into sampling bottles or large volume (100–200 L) samples were taken using dead-end ultrafiltration (DEUF) method [33]. After the water sampling, sodium thiosulphate was used to inactivate the chlorine from the samples during the transport prior to microbiological analyses. In the DEUF method, water samples were collected using ASAHI Rexeed-25A (Asahi Kasei Medical Co., Ltd., Tokyo, Japan) ultrafilters with an average flow rate of 3 L/min. Backflush of the ultrafilters was performed with 500 mL of backflush solution (0.5% Tween 80, 0.01% sodium polyphosphate and 0.001% Y-30 antifoam emulsion). The secondary concentration of DEUF eluates was performed by filtration through Millipore Express PLUS membrane filters (outbreak I, pore size 0.22 μm, Merck KGaA, Darmstadt, Germany) or Nuclepore polycarbonate (PC) filters (outbreak II, pore size 0.4 μm, Whatman, Kent, UK) and/or polyethylene glycol (PEG) precipitation (Table S1). In PEG precipitation, the sample (pH 7–7.5) was mixed with 1% BSA (only for drinking water samples), 0.9 M NaCl and 12% PEG8000 and kept for at least 2 h at 4 °C. After incubation, the sample was centrifuged 10 000× g for 30 min at 4 °C and the pellet was suspended in PBS. Biofilm from water meters was detached and collected as previously described [34]. Before further analyses, all biofilm samples were sonicated for 1 min in 40 kHz (Branson Ultrasonics, Danbury, USA). Biofilm samples were concentrated by filtration through PC filters and PEG precipitation of the filtrate.

#### 2.2.2. Detection of Enteric Virus Genomes

Enteric viruses were analyzed in raw water and drinking water samples either with low volume (1–2 L) adsorption-elution methods or a large volume DEUF method. Low volume samples were concentrated using disc filters (Sartolon polyamide, Sartorius, Göttingen, Germany; Zetapor, Amf-Cuno, Meriden, USA or Nanoceram, Argonide, Sanford, USA) as previously described [35] or modified from Maunula et al. [36], Schultz et al. [37] and Kim and Ko [38]. Samples from contamination sites were analyzed from a volume of 400 mL by PEG precipitation (outbreak I) or extracted directly from a volume of 2.5 mL (outbreak II). Viral RNA and DNA were extracted from the low volume concentrates using the High Pure Viral RNA Kit and High Pure Viral Nucleic Acid Kit (Roche Diagnostics GmbH, Mannheim, Germany), respectively, or the Nuclisens Minimag system (bioMerieux, Marcy-l’Etoile, France). In addition, High Pure Viral Nucleic Acid Large Volume Kit (Roche Diagnostics GmbH) was used with PEG precipitates and directly extracted samples. A sewage sample from municipal wastewater treatment plant was directly treated with Nuclisens kit and the nucleic acid was further purified using OneStep™ PCR Inhibitor Removal (Zymo Research, Irvine, USA). Extractions were made according to the manufacturers’ instructions. Extracted nucleic acids were stored at −75 °C.

For noroviruses, the real-time RT-qPCR assays were carried out in one step, separately for genogroups I and II, using the TaqMan® Fast Virus 1-Step Master Mix (Thermo Fisher Scientific, Austin, TX, USA) as well as primers and probes as previously described [35,39]. For sapoviruses, the real-time RT-qPCR assays were carried using the same protocol with noroviruses [39] or using the QuantiTect probe RT-PCR kit (Qiagen, Hilden, Germany) with a slightly modified norovirus protocol [40]. Sapovirus primers and probes were according to the study by Oka et al. [41] or van Maarseveen et al. [42]. Adenoviruses were detected using primers and a probe described by Jothikumar et al. [43] with the real-time qPCR assay as described previously [44]. The adenovirus real-time qPCR program was 95 °C for 10 min, followed by 45 cycles at 95 °C for 15 s and 60 °C for 1 min. The virus assays were carried out using the QuantStudio 6 Flex Real-Time PCR System (Applied Biosystems, Foster City, USA) or the RotorGene PCR cycler (Qiagen). Quantification of genome copies (GC) of each virus was done using standard curves generated with serially diluted gBlocks® Gene Fragments (Integrated DNA Technologies, Leuven, Belgium). The standard curves were included in each run.

The quality of virus extraction was controlled by positive and negative process controls through all stages of the analytical steps. Spiked mengovirus strain VMC0 and human adenovirus 40 (ATCC VR-931) were used as a positive process controls and sterile deionized water as a negative process control. External amplification controls (EACs) were used to control norovirus GI and GII RT-PCR inhibition in samples as previously described [35]. No inhibition was detected in samples tested negative in norovirus analysis.

Genotyping of sapovirus and norovirus was performed with conventional RT-PCR using One-Step RT-PCR kit (Qiagen). Sapovirus genome was amplified using primers p289 and p290 [45]. Norovirus RNA was amplified in polymerase region A according to Vinjé et al. [46]. The amplicons visualized in gel electrophoresis were sent to Sanger sequencing in the Institute of Biotechnology. Sequences were assigned using the Norovirus Genotyping Tool [47] or with NCBI database using BLAST (basic local alignment search tool).

#### 2.2.3. Enumeration of Indicator Bacteria

Standard methods were used to enumerate *E. coli*, coliform bacteria, intestinal enterococci, and *C. perfringens* count from water and biofilm samples. In brief, *E. coli* and coliform bacteria were analyzed using membrane filtration with LES Endo medium [48] and Chromocult Coliform Agar medium [49] or by using the most probable number (MPN) method based on Colilert-18 QuantiTray [50]. The counts of intestinal enterococci were analyzed using the membrane filtration on Slanetz and Bartley medium [51] or Enterolert (IDEXX Laboratories Inc, Westbrook, USA). Vegetative cells and spores of *C. perfringens* were enumerated on tryptose sulfite cycloserine agar following the international standard [52].

#### 2.2.4. Detection of Microbial Source Tracking (MST) Markers

MST markers were analyzed from nucleic acids extracted from samples of raw water, drinking water and biofilms of water meters either using DEUF method or PC filters. Samples from contamination sites were extracted directly. The nucleic acids were extracted using Chemagic DNA Plant kit (Perkin Elmer, Waltham, USA). Complementary DNA was synthesized as previously described (outbreak I) [34] or by using Superscript IV VILO (outbreak II, Thermo Fisher Scientific, Waltham, USA). MST markers (GenBac3 and HF183) were quantified using DNA-based qPCR assays and RNA-based RT-qPCR assays as described earlier by Pitkänen et al. [27]. The assays were carried out with the QuantStudio 6 Flex Real-Time PCR System (Applied Biosystems) using standard curves generated with serially diluted gBlocks® Gene Fragments (Integrated DNA Technologies).

#### 2.2.5. Detection of Bacterial Pathogens

The presence/absence of thermotolerant *Campylobacter* spp. was determined using culture-based selective enrichment methods following the principles of the international standard [53]. Pathogenic *E. coli* strains (ETEC, EPEC, EHEC, and EAEC) were analyzed from nucleic acid aliquots with in-house PCR method in a clinical laboratory [54].

#### 2.2.6. Detection of Protozoans

In outbreak I, the aliquots of nucleic acids extracted with the Nuclisens Minimag system or Chemagic DNA Plant kit were sent to protozoan (*G. lamblia, E. histolytica, Cryptosporidium* spp. *and D. fragilis*) analysis in the United Medix Laboratories Ltd. In outbreak II, *Giardia* spp. and *Cryptosporidium* spp. were analyzed from drinking water with qPCR using primers and probes described in Hill et al. [55] and Jothikumar et al. [56], respectively, from nucleic acid subsamples. Samples from the contamination site and water meter biofilms were analyzed using the immunomagnetic separation method (IMS) based on standard ISO 15553 [57]. In brief, the sample was centrifuged (15 min, 1100 g) and IMS (Dynabeads G/C Combo, IDEXX laboratories Inc) was done for pellet in the volume of 10 ml. Samples were stained with FITC and DAPI (EasyStain, bioMerieux) and analyzed with epifluorescence microscopy. 

## 3. Results

### 3.1. Clinical Findings

Sapoviruses were found from patients’ stool samples in both outbreaks (Table 1). In outbreak II, sapovirus GIV was detected in one patient sample subjected for sequencing. Moreover, adenoviruses were detected in outbreak I and noroviruses and astroviruses in outbreak II. Noroviruses were not detected in outbreak I. In outbreak II, noroviruses were detected more frequently than sapoviruses. Twelve of the sixteen norovirus positive samples were sequenced successfully and identified as genotypes GI.P7 (n = 11) and GI.P6 (n = 1). In addition, seven out of 16 norovirus positive samples were further analyzed by the genogroup-specific real-time RT-PCR. Of these samples, norovirus GI was detected in all seven samples and norovirus GII in one of seven samples. Sporadic bacterial infections (outbreak II) and *D. fragilis* (outbreak I) were also found in patient samples.

### 3.2. Environmental Investigations

In outbreak I, only *E. coli* and coliform bacteria were analyzed before the start of the chlorination and were detected in one of the two water samples (Table 2). In addition, low counts of coliform bacteria were detected in three out of 91 water samples taken after chlorination on 17th October 2016 and 19th October 2016, and two out of nine biofilm samples on 28th November 2016. Water samples were collected for pathogen and MST-marker analyses only after chlorination. Traces of GenBac3 rRNA were found from one of the three samples on 28th November 2016. In the sample taken from the contamination site, high numbers of both pathogens and indicators were detected. Typing of sapovirus was unsuccessful for contamination site sample. The raw water sample was positive only for GenBac3 rRNA and coliform bacteria. Sewage sample taken from the municipal wastewater treatment plant on 26th October 2016 was positive for sapovirus (genotype GI.2, accession number MK689409) and *D. fragilis*.

In outbreak II, samples were taken before and after the start of the chlorination. Low *E. coli* and intestinal enterococci counts as well as both MST markers (GenBac3 and HF183) were detected from the water before chlorination (Table 3). In a sample taken after the start of the chlorination, sapovirus and genes of enteropathogenic *E. coli* (EPEC) were detected from the drinking water. Sapovirus genotyping was attempted but failed most probably due to the small number of viruses in the sample. Findings of fecal microbes in drinking water, however, led to the decision to perform intensive chlorination. After intensive chlorination, intestinal enterococci were detected in two out of 48 water samples taken from the same site on 15th February and 22nd February 2018. Also, small numbers of GenBac3 rDNA and rRNA copies were detected in three water samples on 15th February 2018. The sample taken from the contamination site on 31st January 2018 contained the same pathogens than detected from the patients and high levels of fecal indicators. Typing of sapovirus was unsuccessful for the contamination site sample. A biofilm sample from water meter on 31st January 2018 was positive only for GenBac3 rDNA and rRNA.

## 4. Discussion

This study presents two waterborne outbreaks caused by drinking water pipe breakage and subsequent contamination of the distribution network. The sudden onset of symptoms and clinical picture of the illness fitted symptoms of viral infection [12]. Stool samples collected from patients confirmed that most of the clinical cases were due to enteric virus infections and sapoviruses were found from patients’ samples in both outbreaks. Sapovirus genotype GI.2 was detected from a sewage sample in outbreak I and sapovirus GIV in a one patient sample in outbreak II. Genotype GI.2 is one of the predominant genotypes worldwide and sapovirus GIV predominated in several countries in 2007 [12]. Unfortunately, patient samples were not sequenced more comprehensively to determine sapovirus genotypes. In many countries, including Finland, norovirus has been the most common causative agent in waterborne outbreaks [5,6], while the linkage of sapovirus infections to possible waterborne spread and outbreaks is rare [9,10]. To our knowledge, this is the first outbreak study worldwide describing the detection of sapovirus in drinking water. In the future, the significance of this emerging virus may increase and thus testing for sapovirus is important to include in waterborne outbreak investigations.

In both outbreaks, untreated municipal wastewater entered into the drinking water distribution network. Raw wastewater reflects the infection burden among the population and can contain a wide variety of pathogens. Water samples taken from the contamination sites contained the same pathogens that were detected from patient samples. However, of these pathogens only sapovirus and EPEC were detected in drinking water in outbreak II. In outbreak I, no water samples were obtained for pathogen analyses before start of the chlorination, which is presumably the main reason behind the non-detection of pathogens from drinking water. However, the first samples taken before chlorination in outbreak I were positive for coliform bacteria indicating the deficiency in the water quality. In outbreak investigations, it is important to collect enough water before mitigation actions for possible future use, in this case e.g., for sapovirus analysis. However, the pathogen sampling should not delay the actions necessary to prevent further spread of infections.

Overall, pathogens are not analyzed as comprehensively as fecal indicator bacteria (*E. coli* and intestinal enterococci) in environmental investigations of outbreaks. This is partly due to their higher cost compared to indicator analyses and the need for expert laboratories to conduct the tests. Even though FIB has often been insufficient to prove the safety of water [58,59,60,61], in this study, these indicators were able to detect the water contamination in both outbreaks. In outbreak I, coliform bacteria and in outbreak II, coliform bacteria, *E. coli*, intestinal enterococci, and *C. perfringens* were detected in drinking water. Noteworthy, sporadic findings of intestinal enterococci were detected in water even after intensive chlorination in outbreak II. These findings support the use of traditional FIB in water quality assessments during outbreak investigation. However, the value of indicators in the prediction of water contamination seems to be case-specific and may require massive contamination as was the situation in the outbreaks described herein and in previous outbreaks described by Kauppinen et al. [35]. 

In this study, the suitability of molecular qPCR assays for fecal source tracking markers (HF183 and GenBac3), along with the traditional FIB was evaluated during waterborne outbreak investigations. The use of genetic source identifiers may provide more sensitive detection of the contamination especially when the assays are targeted to rRNA transcripts in addition to the rDNA [27]. Further, by using a host-specific marker, such as HF183 it is possible to identify the source of the contamination. In contamination sites, HF183 and GenBac3 numbers were comparable or higher than the numbers of pathogenic viruses. Moreover, the markers targeting to host-specific sequences from *Bacteroidales* clearly outnumbered traditional FIB in contamination site samples and thus could be considered for use as specific and sensitive fecal indicators of drinking water quality. Particularly, the human-specific marker HF183 showed promising results and the findings in water were in concordance with pathogen findings. On the other hand, GenBac3 prove to be a very sensitive marker and small GenBac3 copy numbers were found in drinking water after chlorination in both outbreaks and even after intensive chlorination in outbreak II. Interestingly, Diston et al. [62] found in a Swiss groundwater study that genetic markers of *Bacteroidales* are sensitive indicators, but due to the higher presence of these markers compared to enteric viruses may overestimate the risk from enteric viral pathogens. Thus, more data is needed for the correct interpretation of the significance of GenBac3 marker detection after intensive chlorination in terms of health risk assessment.

Mitigation actions, including boil water advisory, providing an alternative drinking water source and chlorination of the drinking water network, were conducted in both outbreaks and proved efficient in controlling the outbreaks. Previous studies have shown the long persistence of enteric viruses and protozoans in drinking water distribution systems in cases without proper treatment or removal of the contamination source [35,63,64,65]. Even though chlorine has been shown to be an efficient decontaminant in the drinking water distribution system [65], the possible stagnant locations (i.e., dead-ends) in the network and deposits accumulated on the inner surfaces of the old pipes may hamper the success of the chlorination. These factors may explain the sporadic microbial findings in water samples followed chlorination. Therefore, it is important to allow sufficient time for chlorination and to ascertain the purity of the water with microbiological analyses as was carried out in these outbreaks. The aging water infrastructure [66] and improper drinking water pipeline construction practices pose a major challenge for water supply and may compromise drinking water safety even more often in the future.

*D. fragilis* detection from patient samples induced media headlines and health concerns among the water consumers. The questionable pathogenesis of this parasite [67,68] initiated a more throughout epidemiological investigation (unpublished results). Lack of knowledge related to the drinking waterborne transmission of *D. fragilis* increased the uncertainty of crowds and up kept the media attention on the topic over a prolonged time.

## 5. Conclusions

To our knowledge, this is the first outbreak study describing the detection of sapovirus in drinking water. Further, herein we proved the suitability of source tracking identifiers to be applied in waterborne outbreak investigation along with pathogens and water quality indicator analyses. Main conclusions are as follows:

- This study highlights the importance of sapovirus as a waterborne pathogen, and warrants the need for testing of multiple pathogens during outbreak investigation
- The MST markers proved useful in the detection of contamination and especially HF183 findings were in concordance with the pathogen results, supporting its use in drinking water outbreak investigations
- Boil water advisory, alternative drinking water source and chlorination were effective mitigation actions during the outbreaks
- The role of *D. fragilis* as human pathogen and its drinking waterborne transmission potential requires further studies

## Figures and Tables

**Table 1:** The number of positive patient samples/no. of studied samples.

Microbe	Outbreak I	Outbreak II
Sapovirus ^1^	16/31	11/33
Norovirus ^1^	0/31	16/33
Adenovirus ^1^	3/31	0/33
Astrovirus ^1^	0/31	7/33
Rotavirus ^1^	0/31	0/33
ETEC ^1^	Nd ^3^	2/34
EHEC ^1^	Nd	1/34
*Plesiomonas shigelloides* ^2^	0/37	1/34
*Yersinia* spp., *Salmonella* spp., *Campylobacter* spp. ^2^	0/37	0/34
*Cryptosporidium* spp., *Giardia lamblia*, *Entamoeba histolytica* ^1^	0/5	0/38
*Dientamoeba fragilis* ^1^	2/5	Nd

^1^ Analyzed using PCR/RT-PCR; ^2^ Analyzed using cultivation; ^3^ Nd, not done.

**Table 2:** Occurrence (the number of positives/no. samples) and counts (genome copies (GC), most probable number (MPN) or colony forming units (CFU)/100 mL) of studied microbes in water, biofilm, sewage and contamination site samples in outbreak I.

Microbe	Raw Water	Water Before Chlorination	Water After Chlorination	Biofilms	Sewage	Contamination Site
Sapovirus	0/1	Nd ^1^	0/7	0/9	1/1	1/1 (1.0 × 10^7^)
Adenovirus	0/1	Nd	0/3	0/9	Nd	1/1 (5.0 × 10^6^)
*Giardia lamblia*, *Entamoeba histolytica*, *Cryptosporidium* spp. and *Dientamoeba fragilis*	0/1	Nd	0/7	0/9	*Dientamoeba fragilis*	*Dientamoeba fragilis*
GenBac3 (rDNA)	0/1	Nd	0/3	0/9	Nd	1/1 (4.5 × 10^7^)
GenBac3 (rRNA)	1/1 (8.0 × 10^0^)	Nd	1/3 (7.0×10^0^)	0/9	Nd	1/1 (7.5 × 10^8^)
HF183 (rDNA)	0/1	Nd	0/3	0/9	Nd	1/1 (6.3 × 10^6^)
HF183 (rRNA)	0/1	Nd	0/3	0/9	Nd	1/1 (2.7 × 10^7^)
*E. coli*	0/1	1/2	0/91	0/9	Nd	1/1 (2.8 × 10^5^)
Coliform bacteria	1/1 (3.0 × 10^−2^)	1/2	3/91 (1.0 – 4.0×10^0^)	2/9 (1.0 – 2.0 × 10^1^)	Nd	1/1 (1.2 × 10^6^)
Intestinal enterococci	0/1	Nd	0/29	0/9	Nd	Nd
*C. perfringens*	0/1	Nd	0/12	0/9	Nd	1/1 (2.0 × 10^4^)

^1^ Nd, not done.

**Table 3:** Occurrence (the number of positives/no. samples) and counts (GC, MPN or CFU/100 mL) of studied microbes in water, biofilm and contamination site samples in outbreak II.

Microbe	Water before Chlorination	Water after Chlorination	Water after Intensive Chlorination	Biofilms	Contamination Site
Sapovirus	0/1	1/2 (<LOQ ^2^)	0/3	0/1	1/1 (1.9 × 10^5^)
Norovirus GI	0/1	0/2	Nd	0/1	1/1 (2.3 × 10^5^)
Norovirus GII	0/1	0/2	Nd	0/1	1/1 (2.8 × 10^3^)
Adenovirus	0/1	0/1	Nd	Nd	0/1
ETEC, EPEC, EHEC and EAEC ^3^	0/1	1/2 (EPEC)	0/3	0/1	1/1 (EHEC, ETEC, EAEC)
*Campylobacter* spp.	0/1	0/2	Nd	Nd	0/1
*Giardia* spp. and *Cryptosporidium* spp.	0/1	0/2	0/3	0/1	0/1
GenBac3 (rDNA)	1/1 (5.9 × 10^3^)	1/2 (6.6 × 10^3^)	3/3 (<LOQ)	1/1 (3.1 × 10^2^)	1/1 (6.3 × 10^10^)
GenBac3 (rRNA)	1/1 (4.4 × 10^5^)	1/2 (1.9 × 10^5^)	3/3 (<LOQ)	1/1 (1.7 × 10^4^)	1/1 (1.1 × 10^13^)
HF183 (rDNA)	1/1 (7.7 × 10^2^)	1/2 (9.0 × 10^2^)	0/3	0/1	1/1 (6.1 × 10^9^)
HF183 (rRNA)	1/1 (5.1 × 10^3^)	1/2 (3.8 × 10^2^)	0/3	0/1	1/1 (8.0 × 10^12^)
*E. coli*	1/5 (2.0 × 10^0^)	0/19	0/48	0/1	1/1 (5.5 × 10^5^)
Coliform bacteria	1/5 (1.2 × 10^1^)	1/19 (3.0 × 10^0^)	0/48	0/1	1/1 (1.7 × 10^6^)
Intestinal enterococci	2/5 (1.0 – 2.0 × 10^0^)	2/19 (1.0–2.0 × 10^0^)	2/48 (4.0 × 10^−1^ – 1.0 × 10^0^)	0/1	1/1 (9.0 × 10^4^)
*C. perfringens*	Nd ^1^	1/9 (1.0 × 10^−1^)	0/22	0/1	1/1 (5.9 × 10^3^)

^1^ Nd, not done; ^2^ LOQ, limit of quantitation; ^3^ Qualitative analysis.

## Supplementary Materials

The following are available online at https://www.mdpi.com/1660-4601/16/22/4376/s1, Table S1: Secondary concentration procedures for target microbes analyzed with the DEUF method.
