# Supplementary material for: Two Drinking Water Outbreaks Caused by Wastewater Intrusion Including Sapovirus in Finland
Source: Int J Environ Res Public Health. 2019 Nov 9;16(22):4376. doi: 10.3390/ijerph16224376 (PMC6888097; doi:10.3390/ijerph16224376)
Supplement: Supplementary file 1 [file ijerph-16-04376-s001.pdf]

Table S1. Secondary concentration procedures for target microbes analyzed with the DEUF method.

**Secondary concentration procedure**	**Target microbes**	**Outbreak**
Direct analysis of DEUF eluate	*E. coli*, coliform bacteria, *Clostridium perfringens*	Outbreak I
Millipore Express PLUS membrane filtration	*Giardia lamblia, Entamoeba histolytica, Cryptosporidium* spp. and *Dientamoeba fragilis*, GenBac3 (rDNA), GenBac3 (rRNA), HF183 (rDNA), HF183 (rRNA)	Outbreak I
PEG precipitation after Millipore Express PLUS membrane filtration	Sapovirus, adenovirus	Outbreak I
Direct analysis of DEUF eluate	*Clostridium perfringens*	Outbreak II
Nuclepore polycarbonate (PC) filtration	ETEC, EPEC, EHEC and EAEC, *Giardia* spp. and *Cryptosporidium* spp., GenBac3 (rDNA), GenBac3 (rRNA), HF183 (rDNA), HF183 (rRNA)	Outbreak II
Direct PEG precipitation	Sapovirus	Outbreak II
